# Point-of-Care Ultrasound: A Multimodal Tool for the Management of Sepsis in the Emergency Department

**DOI:** 10.3390/medicina59061180

**Published:** 2023-06-20

**Authors:** Effie Polyzogopoulou, Maria Velliou, Christos Verras, Ioannis Ventoulis, John Parissis, Joseph Osterwalder, Beatrice Hoffmann

**Affiliations:** 1 Emergency Medicine Department, Attikon University Hospital, 12462 Athens, Greece; effiepol@med.uoa.gr (E.P.); christos.verras@gmail.com (C.V.); jparissis@yahoo.com (J.P.); 2 National Centre of Emergency Care (EKAB), 11527 Athens, Greece; 3 Department of Occupational Therapy, University of Western Macedonia, 50200 Ptolemaida, Greece; iventoulis@uowm.gr; 4 Polipraxis, 9000 St. Gallen, Switzerland; jo@j-osterwalder.ch; 5 Department of Emergency Medicine BIDMC, One Deaconess Rd, WCC2, Boston, MA 02215, USA; bhoffma2@bidmc.harvard.edu

**Keywords:** ultrasound, sepsis, emergency department, point-of-care, POCUS

## Abstract

Sepsis and septic shock are life-threatening emergencies associated with increased morbidity and mortality. Hence, early diagnosis and management of both conditions is of paramount importance. Point-of-care ultrasound (POCUS) is a cost-effective and safe imaging modality performed at the bedside, which has rapidly emerged as an excellent multimodal tool and has been gradually incorporated as an adjunct to physical examination in order to facilitate evaluation, diagnosis and management. In sepsis, POCUS can assist in the evaluation of undifferentiated sepsis, while, in cases of shock, it can contribute to the differential diagnosis of other types of shock, thus facilitating the decision-making process. Other potential benefits of POCUS include prompt identification and control of the source of infection, as well as close haemodynamic and treatment monitoring. The aim of this review is to determine and highlight the role of POCUS in the evaluation, diagnosis, treatment and monitoring of the septic patient. Future research should focus on developing and implementing a well-defined algorithmic approach for the POCUS-guided management of sepsis in the emergency department setting given its unequivocal utility as a multimodal tool for the overall evaluation and management of the septic patient.

## 1. Introduction

Sepsis is a potentially life-threatening condition that occurs when the immune system of the host mounts an inappropriate inflammatory response to infection. As a result of the overt and dysregulated host response to infection, tissue damage ensues followed by organ failure and sometimes death. Even though sepsis does not always have a fatal outcome, lifelong disabilities occur quite often in survivors [1]. In a recent analysis of the Global Burden of Disease Injuries and Risk Factors [2], sepsis-associated morbidity was estimated at 48.9 million global cases, while the mortality rate was reported at approximate 11 million deaths, or one in five deaths worldwide. Of the 48.9 million cases, 33.1 million were attributed to underlying infections, whereas the remaining 15.8 million were due to underlying injuries or non-communicable diseases. Although sepsis represents one of the major causes of the total health burden in the emergency department (ED), its incidence remains underestimated, mainly owing to the fact that it is an intermediate and not a primary cause of illness and death [2,3]. In the forthcoming years, its rate is expected to increase further due to population aging coupled with a constant rise of antibiotic-resistant bacteria and an increase in the number of immunocompromised patients. Thus, in accordance with the statement “Time is tissue”, early identification and prompt initiation of treatment are of paramount importance and are positively correlated with favourable outcomes [1].

Point-of-care ultrasound (POCUS) is a well-established and widely used imaging modality in emergency medicine [4]. Regarding sepsis, growing evidence has shown that POCUS integration to the management of sepsis in the ED favours a tailored approach and allows serial clinical assessments during resuscitation [5]. A recent literature review summarizes the well-recognized role of POCUS for septic patients in the ED and points out the absence of large-scale studies [6]. Despite this evidence, the use of POCUS is not included in the latest Surviving Sepsis Campaign (SSC) guidelines [1]. A plausible explanation could be the lack of well-established and validated protocols for patients with sepsis or septic shock, as opposed to widely used POCUS protocols for undifferentiated shock (e.g., rapid ultrasound in shock—RUSH protocol) [7], dyspnoea (bedside lung ultrasound in emergency—BLUE protocol) [8,9], major cardiac pathologies (focus cardiac ultrasound—FoCUS) [10], acute heart failure (e.g.,rapid cardiothoracic ultrasound—CaTUS protocol) [11], advanced cardiac life support (echo in life support—ELS) [12], etc.

Considering that sepsis-related morbidity and mortality rates remain high, detection and exploitation of enhanced bedside techniques that would facilitate the early diagnosis and effective management of sepsis becomes imperative. The aim of this manuscript is to highlight and define the role of POCUS as an integrated multimodal tool for the bedside evaluation, diagnosis, monitoring and treatment of the septic patient in the ED setting.

## 2. POCUS as a Multimodal Tool

**Figure 1:**
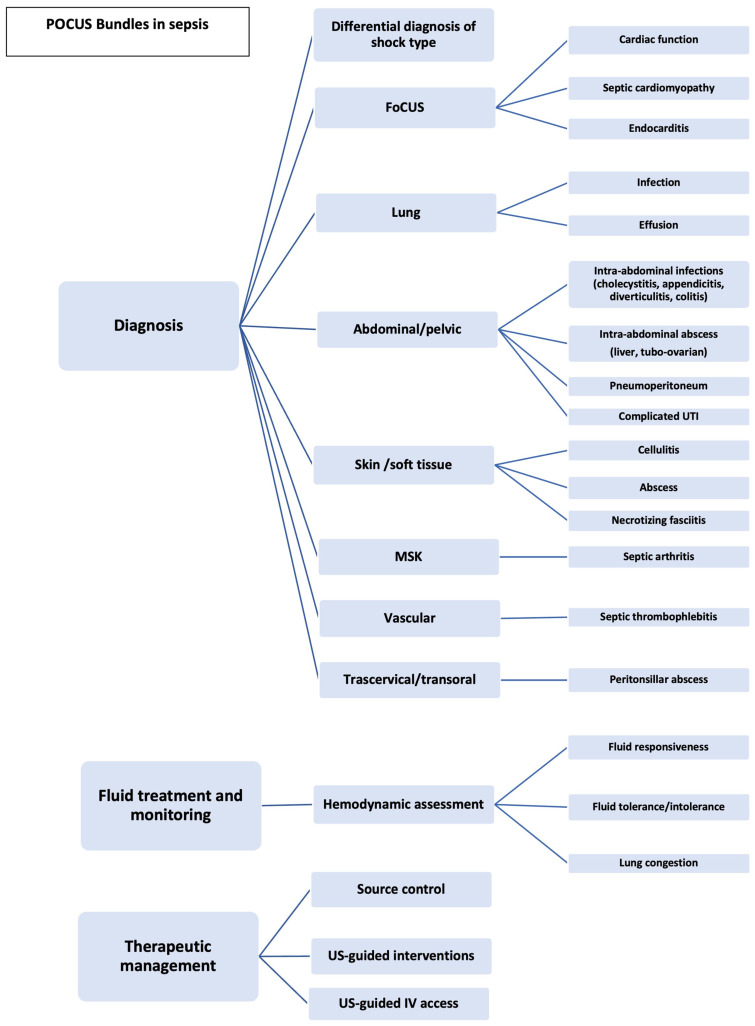
POCUS bundles in sepsis. Abbreviations: FoCUS: focused cardiac ultrasound; IV: intravenous; MSK: musculoskeletal; POCUS: point-of-care ultrasound; US: ultrasound; UTI: urinary tract infection.

The multimodal use of POCUS in the management of the septic patient in the ED, the applications, the relevant queries and findings are analysed further down. A comprehensive and step-by-step approach is illustrated in Figure 1.

## 3. POCUS as a Tool for Diagnosis

**Differential diagnosis of the type of shock.** Studies regarding the contribution of POCUS in the differential diagnosis of critically ill patients presenting in the ED with undifferentiated hypotension have yielded controversial results due to the moderate sensitivity of POCUS in excluding any type of shock with the exception of obstructive. At the same time, POCUS has high specificity (>91% for the four subtypes of nontraumatic hypotensive shock and approximately 80% for mixed types) [13,14]. Moreover, agreement (as measured by Kappa coefficient—k) between initial diagnosis—based on both clinical and POCUS evaluation—and final diagnosis ranges from 0.717 to 1.00 (*p* < 0.003) [15,16,17]. The main downside with regard to the differential diagnostic value of POCUS for distributive/septic shock lies in the complex pathophysiology of sepsis and the lack of distinct pathognomonic sonographic findings. However, studies have shown that the integration of POCUS into the initial evaluation of undifferentiated shock can effectively narrow down the spectrum of initial differential diagnosis and thus accelerate the recognition of the type of shock [13,14,18,19]. In the event that sepsis is being suspected, multi-organ POCUS may further help the clinician reach a certain diagnosis and identify the septic cause.

**Focused Cardiac Ultrasound.** Focused cardiac ultrasound (FoCUS)provides a rapid, but gross, assessment of cardiac anatomy, cardiac function and systemic haemodynamics. It permits the identification and/or exclusion of life-threatening causes of haemodynamic failure, the evaluation of cardiac contractile activity and the recognition of coexisting pathologies such as heart failure [10]. Specifically, FoCUS includes the evaluation of left ventricular (LV) and right ventricular (RV) size and function, the eyeball estimation of ejection fraction (EF), the assessment of gross abnormalities of the mitral, aortic and tricuspid valves, as well as the assessment of regional wall motion abnormalities. Of note, a recent study demonstrated that the incidence of RV and LV dysfunction is particularly high in septic patients (48% and 63%, respectively) [20]. Additional features involve the identification of pericardial effusion and RV enlargement, as well as the assessment of fluid responsiveness and fluid tolerance/intolerance. Other special attributes may entail the recognition of sonographic findings consistent with septic cardiomyopathy and the differentiation of other entities such as diastolic dysfunction, ischemic cardiomyopathy or non-ischemic stress-induced cardiomyopathy (Takotsubo cardiomyopathy) [5,21]. Even though transesophageal echocardiography is the gold standard, transthoracic echocardiography may also be used in the ED setting for the detection of vegetations in cases of suspected infective endocarditis [22]. In addition, the ultrasound (US) assessment of the inferior vena casa (IVC) diameter and the collapsibility index provides further insight into haemodynamics and volume status and right atrial pressure [23]. Recent focus has been given on the trans-hepatic approach to IVC as an alternative method when subcostal view is not feasible [24]. A retrospective study revealed that the use of echocardiography in this group of patients is correlated with a significant improvement in 28-day mortality [25]. Notably, FoCUS can potentially change the therapeutic plan in more than 50% of septic patients [26].

**Lung Ultrasound.** One of the most commonly detected sources of sepsis is pulmonary tract infection [27]. Lung US is a well-established and extremely useful bedside diagnostic imaging modality for the septic patient. Available evidence indicates a clear superiority of lung US as a screening tool over chest X-ray in terms of identifying various pulmonary conditions. The main indication of POCUS is the detection of pneumonia by visualizing focal B-lines, lung consolidation or hepatization, dynamic air bronchograms, subpleural abnormalities and/or irregular and thickened pleura [28,29,30]. A meta-analysis of 10 studies demonstrated that POCUS had a sensitivity of 95% and a specificity of 93% in diagnosing pneumonia, compared to chest X-ray which had a sensitivity of 69% and a specificity of 77% [31]. Moreover, POCUS can detect parapneumonic effusions anddifferentiate between uncomplicated pleural effusions and empyema. A meta-analysis of 12 studies showed that the sensitivity and specificity of US for the diagnosis of pleural effusion were 94% and 98%, respectively, while the sensitivity and specificity of chest radiography were 51% and 91%, respectively [32].

**Abdominal, Pelvic and Urinary Tract Ultrasound.** Abdominal infections including urinary tract infections are one of the most common sources of sepsis [33]. POCUS can be used as an adjunct tool for the diagnosis of several intra-abdominal infections, such as acute cholecystitis, acute appendicitis, diverticulitis and colitis, as well as complicated urinary tract infections (pyelonephritis, hydronephrosis, renal abscess). The sensitivity and specificity of US in detecting gallstones are 89.8% and 88%, respectively [34]. In addition, it has been shown that the absence of gallstones on POCUS performed in the emergency setting can effectively rule out acute cholecystitis [35]. In case of acute appendicitis, POCUS has a sensitivity of 86% and a specificity of 91% [36], whereas renal USseems to have modest diagnostic accuracy for hydronephrosis. A meta-analysis of five studies revealed that the sensitivity and specificity of POCUS in hydronephrosis were 70.2% and 75.4%, respectively, and the specificity was significantly improved (94.4%) for moderate to severe hydronephrosis [37]. POCUS is also considered a rapid and precise imaging modality for the diagnosis of diverticulitis with a sensitivity of 92.7% and specificity of 90.2%. Nonetheless, the sensitivity (50%) is low for complicated diverticulitis [38]. Depending on the operator’s experience, POCUS can detect intra-abdominal abscesses including liver or tubo-ovarian abscesses [39]. Pneumoperitoneum due to visceral perforation and consequent peritonitis can be identified as well. Available data indicates that POCUS is a more sensitive modality compared to X-ray for identifying free air in the abdomen. US has a sensitivity of 93%, a specificity of 64% and a positive predictive value of 97% for the diagnosis of pneumoperitoneum, while radiography has a sensitivity of 79%, a specificity of 64% and a positive predictive value of 96% [40]. Regarding intra-abdominal fluid, POCUS detects even small amounts of ascites that are undetectable by physical examination with overall sensitivity and specificity of more than 90%. It also has significant diagnostic accuracy to distinguish transudate and exudate ascites. The minimum volume of fluid that can be visualized is approximately 100 mL [23,41,42,43].

**Skin and Soft Tissue Ultrasound.** The main use of POCUS is the differentiation between cellulitis and more severe conditions such as abscesses and necrotizing fasciitis [44]. Existing evidence indicates that POCUS has a sensitivity of 97% and a specificity of 83% in identifying abscesses in ED patients with skin and soft tissue infections [45]. In case of necrotizing fasciitis, the overall sensitivity ranges from 85.4 to 100%, while the specificity ranges from 44.7 to 98.2%. Perifascial fluid is considered the most sensitive sign and subcutaneous emphysema seems to be the most specific sign [46]. Furthermore, US can distinguish cellulitis from other conditions with similar clinical appearance, such as deep vein thrombosis (DVT) [45,47].

**Musculoskeletal Ultrasound.** Musculoskeletal infections and, in most cases, septic arthritis are typical sources of sepsis in the ED. US imaging has a sensitivity of 93.4% and a specificity of 100% for diagnosing joint effusion [48]. One of the challenges in identifying septic arthritis is the wide range of potential mimics, including gout, pseudogout, reactive arthritis, rheumatoid arthritis and osteoarthritis. These conditions present with similar symptoms such as joint pain, swelling and stiffness, thus making it difficult to differentiate them from septic arthritis based on clinical examination alone. POCUS can help distinguish septic arthritis from these clinical entities by visualizing the joint space, the synovial membrane and the surrounding soft tissue [49,50,51].

**Vascular Ultrasound.** POCUS seems to be of great utility for the identification of deep vein thrombosis-associated complications, which could potentially serve as the initial nidus of sepsis. The major complication is septic thrombophlebitis, whichis commonly seen in intravenous drug users or patients with indwelling central venous catheters and less frequently in the rather rare clinical entity called Lemierre syndrome [52,53]. In septic thrombophlebitis, US shows a non-compressible vein with an anechoic or echogenic thrombus in its lumen, vessel wall thickening and loss of colour Doppler flow. In case of Lemierre syndrome, an hyperechoic thrombus within the internal jugular vein or other neck or facial veins might be visualized [54].

**Transcervical or Transoral Ultrasound.** POCUS has emerged as a cost-effective and valuable tool with high diagnostic accuracy (sensitivity 90–95% and specificity 95–100%), which can be used as an acceptable alternative to computed tomography (CT) in patients presenting to the ED with a peritonsillar abscess. It provides a real-time imaging of the tonsillar fossa, peritonsillar space and surrounding structures, and can identify the size, the location and the extent of the abscess, as well as the presence of any residual fluid collections [55,56].

Table 1 summarizes the most common diagnoses and the related ultrasound findings in the septic patient.

**Sepsis mimics.** Important diagnostic challenges in the acute care setting are the non-infectious mimics of sepsis, which mandate not only a high level of suspicion but also multiple diagnostic tools and techniques. Sepsis mimics include, among others, diabetic ketoacidosis, Kawasaki disease, anaphylaxis, adrenal crisis, aspiration, heat emergencies, thyroid disease, intestinal ischemia, hypovolemia, withdrawal state, spinal cord injury, vasculitis due to chronic inflammatory autoimmune disorders and toxic ingestion/overdose. These diseases usually present in a same manner as sepsis due to common pathophysiologic response [69,70].

Identifying the source of infection is crucial in distinguishing sepsis from its mimics. A thorough evaluation of the patient’s medical history, physical examination and laboratory tests, coupled with the use of POCUS, can help to identify the underlying cause of the patient’s symptoms and guide appropriate treatment.

## 4. POCUS as a Tool for Fluid Treatment and Monitoring

Sepsis often leads to shock. This is due to 1. hypovolemia, due to venous “pooling” and capillary leak; 2. vasodilation; and 3. cardiomyopathy. Therefore, it is understandable that fluid administration is one of the central pillars of early sepsis treatment. The SSC guidelines recommend that at least 30 mL/kg of intravenous crystalloids should be applied within the first three hours and that for adult patients with sepsis or septic shock, dynamic measurements to guide fluid resuscitation should be preferred over clinical examination or static parameters [1]. These recommendations are considered weak and are, therefore, controversial because the evidence is rated qualitatively low to very low.

Early intravenous fluid therapy with crystalloids seems to be uncontroversial. However, the amount, in which time frame and the moment when the treatment has to be stopped are disputed. Namely, there is good evidence that only about 50% of critically ill patients are fluid responsive; i.e., benefit, and the other half run the risk of being overhydrated [71]. The SSC guidelines of 2021 recommend intravenous fluid boluses or the use of a passive leg raising test in terms of fluid challenges with repetitive measurement of stroke volume, stroke volume variation, pulse pressure variation or echocardiography for this purpose [1]. For a long time, the focus of fluid resuscitation has been on cardiac output or forward flow. Yet, there is increasing evidence in the literature that back flow or venous congestion at a certain point cancels out the benefit of forward flow [71].

In our opinion, POCUS plays a central role by currently becoming the preferred tool for assessing haemodynamics. Considering the Frank–Starling–Sarnoff curve, Jon-Emilie Kelly presents the physiological framework for an ultrasound concept of volume management which uses Doppler ultrasound to gauge cardiac filling and output in addition to inferior vena cava morphology, thus combining the two concepts of fluid responsiveness (increase in stroke volume on volume administration) and tolerance/intolerance (no or deleterious effect on fluid administration) [72]. His model emphasizes the dynamic nature of the cardiac function curve (cardiac output in relation to preload), which shows that when left ventricular performance deteriorates, the relationship between preload and the ability to respond with stroke volume is less clear. Because the ejection fraction does definitely determine preload dependence, predicting the response to additional IV fluid requires dynamic measures [72]. In volume therapy, however, it is not only important to predict fluid responsiveness but also fluid tolerance and intolerance [73]. Unfortunately, there is no validated standard. We believe that combination and comparison of the following sonographic parameters, measured before and after fluid bolus, might prospectively estimate fluid responsiveness and tolerance/intolerance: stroke volume using velocity time integral (VTI), venous parameters such as antero-posterior diameter and respiratory variability of the vena cava inferior, and the Doppler spectrum of the median hepatic vein with focus on the maximum velocity of the diastolic inflow. However, the cut-off values of the differences of the above-mentioned measurands before and after volume administration, which allow us to assume that renewed fluid administration will lead again to fluid responsiveness and fluid tolerance, are missing.

One more comment. The venous excess ultrasound score (VExUS) seems to be unsuitable for fluid therapy of septic shock not only for practical (too complex and technically very demanding inthe emergency situation) but also for conceptual reasons. The VExUS score aims to quantify venous congestion. When providing volume therapy to patients in septic shock, the goal is to predict fluid responsiveness, fluid tolerance and fluid intolerance. For this, we do not need quantification of venous congestion, but it is of paramount importance to know if fluid-responders to further fluid therapy are at risk of venous congestion [74].

In addition, the capillary leak, as one reason for pulmonary oedema, which is not detected by these measures, can be checked with lung ultrasound and B-lines, respectively. If B-lines increase, fluid administration should be stopped for the time being and treatment continued with vasopressors, irrespective of the haemodynamic parameters [75].

## 5. POCUS as a Tool for Therapeutic Management

**Source control.** Early source control has been recognized as a determinant of outcomes in patients with sepsis or septic shock since it contributes to the elimination of the source of infection, the control of ongoing contamination and the restoration of premorbid anatomy and function. Some of the strategies used in source control include the drainage of abscesses, the removal of infected necrotic tissue, the extraction of infected devices and the placement of “ostomies”, such as nephrostomies [1,76]. POCUS has emerged as an efficient and cost-effective bedside tool, which serves as an adjunct for many urgent and emergent procedures that can thus be performed with a lower risk of complications compared to a non-US blind landmark-guided technique [4]. Its major indication involves cases of critically ill, haemodynamically unstable patients, wherein CT-guided procedures for sepsis control are contraindicated. Moreover, POCUS can identify the extent and severity of the infection, which is important in determining the appropriate treatment plan.

**Ultrasound-guided interventions.** Thoracentesis is a commonly performed procedure in a septic patient with a large pleural effusion that is highly suspected to be exudative in nature, or in other words suggestive of the presence of empyema. This procedure enables the removal of a large volume of fluid in order to improve oxygenation, gas exchange, respiratory mechanics and haemodynamic stability, and relieve symptoms of dyspnoea. US is a safe, fast and effective tool to determine the volume of infected pleural effusions and can also monitor the volume of pleural effusion drained and decide when to remove the catheter drainage. Moreover, ultrasound guidance to insert chest tubes increases the success rate and the safety of the procedure, especially in ventilated patients or those with small size effusions [77,78]. It also allows the operator to visualize the needle and surrounding structures and identify the most accessible area of pleural fluid reducing the risk of potential complications (i.e., iatrogenic pneumothorax, puncture site bleeding, chest wall haematoma, haemothorax). An observational study of approximately 60,000 thoracenteses showed that POCUS decreased the risk of pneumothorax by 19% [79,80].

Another indication for the therapeutic use of POCUS is US-guided diagnostic and/or evacuative paracentesis in order to reduce the risk of serious complications, such as abdominal wall haematoma, haemoperitoneum and bowel or other abdominal organ perforation. In the ED setting, diagnostic paracentesis should be performed in all septic patients with cirrhosis and ascites if spontaneous bacterial peritonitis is suspected [81,82].

US-guided drainage as a minimally invasive treatment option is recommended in septic patients with acute obstructive biliary disorders, such as acute cholecystitis and acute cholangitis. Although early cholecystectomy is the first-line therapy in the context of acute cholecystitis, some patients carry multiple comorbidities or are at high surgical risk. In such cases, a successful placement of a cholecystostomy tube by US guidance in order to drain the inflamed gallbladder might serve as a bridge to delayed cholecystectomy or a definitive therapy. Ultrasound guided biliary decompression is also considered an alternative option in patients with acute cholangitis and failed endoscopic retrograde cholangiopancreatography or those with surgically altered anatomy. The overall complication rate of the procedure is low, with the most commonly reported complications being vascular injury and bleeding, bile leak and peritonitis, pancreatitis, and iatrogenicpleural transgression [83,84].

POCUS has also been shown to be effective in the drainage of peritonsillar abscesses; it is actually associated with lower complication rates compared to blind incision and drainage procedures, considering the fact that it provides real-time imaging of the abscess and the arteries and therefore improves the safety of the procedure, especially when the volume of pus is small [56,85].

In pyonephrosis with secondary urosepsis, urgent resolution of hydronephrosis is crucial for the infectious source removal and the prevention of renal dysfunction. Ultrasound-guided placement of a urinary stent or a percutaneous nephrostomy tube is an easy and cost-effective technique with high success rates and minimal complications. Renal or perineal abscesses or urinomas might also require drainage under ultrasound guidance [86,87].

In addition, sonography is an excellent adjunct tool for aspiration of joint fluid in patients with suspected septic arthritis. The direct visualization of the effusion by real-time ultrasonography results in fewer failed attempts and a greater success rate, while at the same time, it increases the likelihood of obtaining a positive culture. Compared to the blind landmark-based approach, it can detect and aspirate even small volume effusions [88].

Last but not least, US serves as a helpful tool offering anatomic information, such as the depth of the ligamentum flavum, the widest interspinous space and spinal abnormalities in cases of difficult or even not-visible landmarks for the performance of lumbar puncture when needed in order to diagnose central nervous system infections. It is estimated that in the ED, approximately 30% of the patients have difficult anatomy with less palpable landmarks and in about 15% of them, the landmark-based approach might be traumatic. POCUS identifies the ideal lumbar spine level and reduces the number of needle insertions and redirections, the time to successful procedure, the risk of traumatic taps and the incidence of complications, such as headache and backache [89,90,91].

**POCUS-guided intravenous access.** Establishing venous access in septic patients is technically challenging and often time-consuming due to diminished physiological reserves, hypovolemia, history of intravenous drug abuse and comorbidities. In this context, using US-guided placement of peripheral or central lines is an easy and non-invasive technique that has been shown to improve the success rate and reduce the number of unsuccessful attempts or other complications, compared to a standard procedure based on landmarks [92,93]. Common sites when performing ultrasound-guided peripheral venous access are the superficial veins of the upper limbs, namely the basilic vein, cephalic vein, median vein of the forearm and median cubital vein [94]. As it concerns central venous catheterization, the access points that are mainly used are the femoral vein, subclavian vein and internal jugular vein [93]. Notably, femoral access has been shown to be at a greater risk of catheter-related infections [95,96] compared to subclavian and internal jugular accesses that seem much safer and are correlated with a lower hazard of severe complications [97,98]. Furthermore, US offers an alternative method for the confirmation of successful intravenous line placement [99] and can rule out pneumothorax much faster than antero-posterior chest radiograph [100].

**Figure 2:**
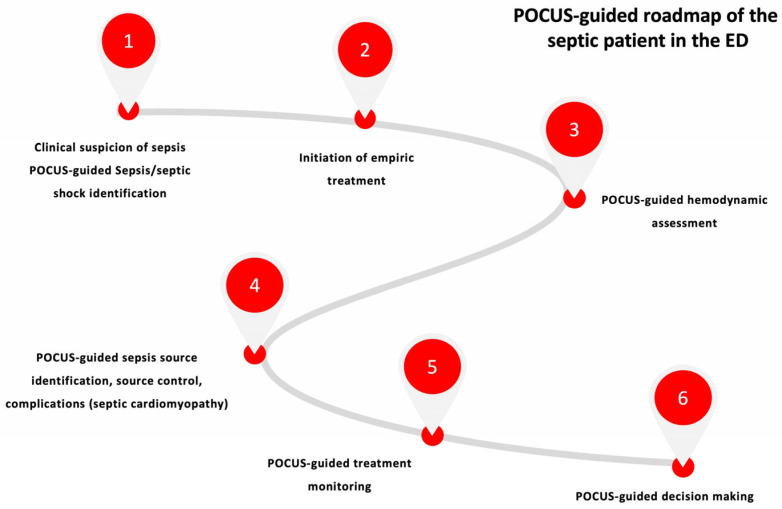
POCUS roadmap of the septic patient in the ED. Abbreviations: ED: emergency department, POCUS: point-of-care ultrasound.

The final step of the roadmap during the septic patient journey in the ED is the “decision making” step that also can be POCUS guided. In this step, ultrasound offers additional information regarding alternative diagnoses, guides escalation/de-escalation fluid and vasopressor therapy. Furthermore, it can be a supportive tool in the final disposition decision making regarding the admission in a common ward or in the intensive care unit (ICU). Figure 2 delineates how POCUS can be incorporated in the initial evaluation, diagnosis and management of patients with sepsis or septic shock in the ED.

It is important to keep in mind that POCUS use should be integrated in the right clinical context and at the right time in order to yield a beneficial rather than a harmful effect [101]. It is a valuable and safe adjunctin addition to some other precise tools including history-taking, physical examination and laboratory tests. However, it has several limitations. It is particularly susceptible to errors since US image acquisitionand interpretation is operator dependent. Preload assessment might also be limited in several cases such as in patients with non-invasive mechanical support, intubated patients or those with intra-abdominal hypertension [23,102].

## 6. Conclusions

POCUS is a useful bedside imaging modality for the initial evaluation of patients with sepsis and septic shock. It represents a substantial time-saving tool which allows for timely diagnosis and prompt initiation of treatment. An US-guided approach in the ED setting can quickly identify the need for a source control strategy, a more targeted antimicrobial therapy and an individualized therapeutic management, therebyimproving outcomes and decreasing sepsis-related mortality. The development of structured algorithms or clinical pathways for the different applications of POCUS, which would incorporate POCUS into ED clinical practice, is of paramount importance and will cover unmet needs regarding the management of the septic patient in the ED.

## Figures and Tables

**Table 1:** The most common diagnoses and the related US findings in the septic patient.

Diagnosis	US Findings
Septic cardiomyopathy [5]	Reduced EF Ventricular hypokinesis
Endocarditis [22]	Amorphous, independent motioned, echogenic masses, associated with cardiac valves Thickened, abnormal valve
Takotsubo cardiomyopathy [57]	Impaired contractility of the apex and hypercontractility of LV base
Severe RV failure [20]	Dilated RV and right atrium
Ischemic cardiomyopathy [26]	RWMAs
Pneumonia [28,29,30]	Focal B-lines Lung consolidation or hepatization Dynamic air bronchograms Subpleural abnormalities Irregular and thickened pleura
Pleural effusion [58]	Anechoic fluid collection above the diaphragm Spine sign Quad sign Sinusoid sign
Empyema [58]	Echogenic pleural effusion ± septations
Cholecystitis [59]	Gallbladder wall thickening (>3 mm) and/or oedema Positive sonographic Murphy sign Pericholecystic fluid Gallstones
Appendicitis [60]	Dilated (>6 mm), aperistaltic, non-compressible appendix Appendicolith Hyperechoic periappendiceal fat
Diverticulitis [61]	Colonic wall thickening >5 mm Fat enhancement Visualized diverticula Air artifacts Tenderness with compression of the probe
Colitis [62]	Increased symmetrical wall thickening Submucosal echogenicity
Intra-abdominal abscess [63]	Anechoic to hypoechoic cystic collections Internal echoes and debris Irregular margins, ±septations
Pneumoperitoneum [64]	Peritoneal stripe enhancement Repeating, horizontal long-path reverberation artifacts that extend into the far field Gas bubbles
Pyelonephritis [65]	Abnormal echogenicity of the renal parenchyma Gas bubbles (emphysematous pyelonephritis) Particulate matter in the collecting system
Cellulitis [44]	Cobble-stone appearance Increased echogenicity of the subcutaneous tissue
Necrotic fasciitis [44]	Fascial and subcutaneous tissue thickening Fascial fluid Subcutaneous air
Skin/soft tissue abscess [66]	Irregular hypoechoic fluid collection
Septic arthritis [67]	Joint effusion Increased peri-synovial vascularity
Septic thrombophlebitis [52]	Venous thrombus Non compressible vein
Peritonsillar abscess [68]	Hypoechoic, heterogeneous or complex cystic structure

Abbreviations: EF: ejection fraction; LV: left ventricular; RV: right ventricle; RWMAs: regional wall motion abnormalities; US: ultrasound.

## Data Availability

Not applicable.
